# Impact of Galvanic Vestibular Stimulation on Anxiety Level in Young Adults

**DOI:** 10.3389/fnsys.2019.00014

**Published:** 2019-04-16

**Authors:** Florane Pasquier, Pierre Denise, Antoine Gauthier, Nicolas Bessot, Gaëlle Quarck

**Affiliations:** Normandie Université, Unicaen, Inserm, Comete, GIP Cyceron, Caen, France

**Keywords:** anxiety, galvanic vestibular stimulation, motion sickness, vestibular system, direct current (DC)

## Abstract

Galvanic vestibular stimulation (GVS) is a non-invasive method used to stimulate the vestibular system. The vestibular system includes the sensors, neural pathways, vestibular nuclei and the cortical areas receiving integrated vestibular inputs. In addition to its role in postural control or gaze stabilization, the vestibular system is involved in some cognitive functions and in emotion processing. Several studies have revealed a modulating effect of vestibular stimulation on mood state, emotional control, and anxiety level. Nevertheless, GVS is known to induce motion sickness symptoms such as nausea. The aim of the present study was to evaluate the tolerability and efficacy of a GVS protocol to be used potentially as a treatment for anxiety, and also to test the impact of stimulation parameters (duration) on anxiety. Twenty-two students underwent three stimulation conditions: (1) a sham session (no stimulation); (2) a single-duration session (38 min of GVS); and (3) a double-duration session (76 min of GVS). Before and after each stimulation, participants completed a Graybiel Scale form for motion sickness symptoms evaluation and a visual analog scale form for anxiety. We observed a significant diminution of anxiety level after a 38-min session of GVS, while a low level of motion sickness was only found following a 76-min session of GVS. Our preliminary study confirms the feasibility of using GVS to modulate anxiety and corroborates the involvement of the vestibular system in the emotional process.

## Introduction

Galvanic vestibular stimulation (GVS) was discovered at the beginning of the 19th century in the context of Alessandro Volta’s experiments (Volta et al., 1918). GVS induces disturbances in equilibrium, and nystagmus through stimulation of the vestibulo-cochlear nerve (Curthoys and MacDougall, 2012). It activates both primary otolithic neurons and primary semi-circular canal neurons (Curthoys and MacDougall, 2012). In GVS, the electrodes are attached to the mastoids behind the ears to stimulate the vestibular afferents, and their cortical projections named vestibular cortex (Dieterich and Brandt, 2008; Utz et al., 2010).

In addition to its role in gaze stabilization and postural control, the vestibular system has an influence on aspects of emotion processing, mood state and mental health (Lopez, 2016; MacDowell et al., 2018). Many studies report a higher probability for patients with vestibular disorder to suffer from depression, panic or any anxiety disorders (Eagger et al., 1992; Mast et al., 2014). Moreover, abnormal asymmetries of vestibulo-ocular reflex were found in major depression population (Soza Ried and Aviles, 2007). Recent studies test the effect of swing protocol or caloric vestibular stimulation on mood state and affective control (Winter et al., 2012; Preuss et al., 2014; Kumar et al., 2016). They report a modulating effect of vestibular stimulation on anxiety level and mood state. Furthermore, anatomical pathways support the link between vestibular system and mental conditions. A review demonstrated that several brain regions are involved in both vestibular signal processing and psychiatric disorders, including the raphe nuclei, locus coeruleus, hippocampus, amygdala, insular cortex, anterior cingulate cortex, putamen, prefrontal cortex, parietal lobe, occipital lobe, and cerebellum (Gurvich et al., 2013).

These researches open new perspectives in the use of vestibular stimulation to improve psychiatric illness, in particular, anxiety disorders. GVS is a variant of transcranial direct current stimulation (tDCS) used to treat depression. GVS and tDCS have several technical advantages: simplicity of use, portability, and the possibility for participants to use them by themselves. Positive effects of tDCS protocols are due to a modification of cortical excitability (Bennabi and Haffen, 2018). In most tDCS studies, electrodes are applied to stimulate the somatosensory cortex or the primary visual cortex but different locations can be used (Utz et al., 2010). This method has a limitation concerning the perimeter of the stimulated zone, because of the large electrode size (Utz et al., 2010). The electric current could diffuse and stimulate other cortical areas, including the brain circuits related to vestibular processing. Moreover, another type of tDCS—the transcutaneous vagal nerve stimulation with electrodes placed behind the ears—has shown promising results in treating major depressive disorders (Fang et al., 2017). This observation is in line with the current diffusion hypothesis.

Despite their technical advantages and the potential effect of GVS on mental disorders, this vestibular stimulation can provoke symptoms of motion sickness such as nausea, vertigo or cold sweating (Quinn et al., 2015). Taking account of this limitation, the primary objective was to demonstrate the tolerability of our experimental protocol almost similar to the stimulation parameters (intensity, duration) used in tDCS treatment (Utz et al., 2010). The second objective was to test the impact of GVS on anxiety. Considering that stimulation parameters (duration, intensity, electrode placements) contribute to the variability of the therapeutic effects of tDCS (Utz et al., 2010; Bennabi and Haffen, 2018), the third objective was to test whether the impact of GVS on anxiety was duration dependent.

## Materials and Methods

Twenty-two students from the University of Caen, Normandy, participated in this study (age: 21.90 ± 1.37 years; 10 females and 12 males). This study was carried out in accordance with the recommendations of CERSTAPS 2019-18-09-37 with written informed consent from all subjects. All subjects gave written informed consent in accordance with the Declaration of Helsinki. The protocol was approved by the CERSTAPS. We excluded individuals with vestibular disorders and those with current or prior psychiatric disorders. One participant stopped the protocol after the first session. For women, experiments were scheduled outside of the menstrual period in order to avoid any bias due to hormonal fluctuations (Dennerstein and Burrows, 1979). The sessions were carried out in the late afternoon, on the same day of the week, and at the same hour to avoid any diurnal or weekly anxiety variations (Haffen and Sechter, 2006), for a total of three randomized sessions, for three consecutive weeks. Before and after each stimulation, participants completed a Graybiel Scale form for motion sickness evaluation (Graybiel et al., 1968), and a 100 mm visual analog scale form (relaxed—tense; Abend et al., 2014) to evaluate mood and psychophysiological alterations (Zealley and Aitken, 1969; Winter et al., 2012, 2013).

Subjects underwent three test sessions: (1) a sham session (no stimulation); (2) a single-duration session (38 min of GVS); and (3) a double-duration session (76 min of GVS). GVS was delivered by a galvanic stimulator (NeuroConn^®^, Germany). Stimulation consisted of 1 mA (Utz et al., 2010) and the polarity was changed four or eight times, depending on the stimulation duration. Anodal stimulation increases cortical excitability (Nitsche and Paulus, 2000). The polarity shifts promote plastic modifications in right and left vestibular cortex. During the stimulation, participants were seated in a quiet room. When stimulation was finished, the participants waited for 10 min to ensure potential neural plasticity before they completed post-tests.

An analysis of variance (ANOVA) was performed on all tests with two repeated factors, session (sham, single-duration, double-duration) and time (pre-tests, post-tests). A Tukey adjusted for multiple comparisons was done as a *post hoc* test. *P*-values ≤ 0.05 were considered statistically significant.

## Results

**Figure 1:**
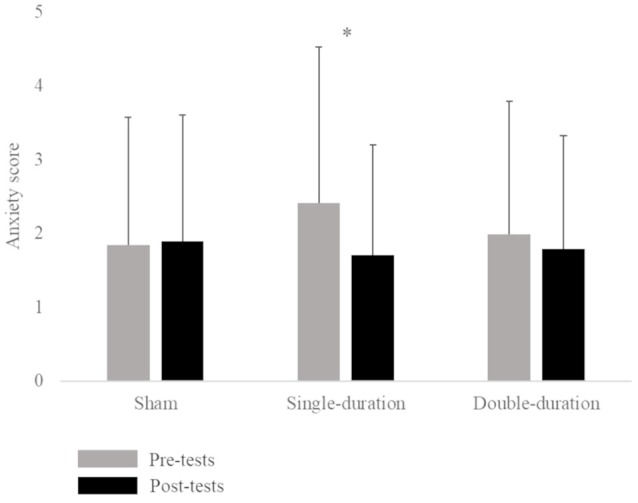
Mean anxiety score (score ± SD) for the three sessions (sham, single-duration, double-duration) for pre-tests and post-tests. *p < 0.05.

The ANOVA for the Graybiel Scale form indicated a significant interaction between session and time factors (*F* = 5.92, *p* = 0.005). The Tukey *post hoc* test demonstrated an increase in motion sickness symptoms between pre-tests (0.48 ± 1.03) and post-tests (1.62 ± 2.20) of the double-duration session. The statistical analysis also revealed a significant interaction between session and time factors for the anxiety score (*F* = 3.70, *p* = 0.033). A *post hoc* test showed a significant decrease between pre-tests (2.41 ± 2.12) and post-tests (1.71 ± 1.49) of the single-duration session (Figure 1; Supplementary Table S1). We did not observe any effect of gender on anxiety. There was no significant interaction of session, time and gender factors (*p* = 0.68).

## Discussion

Our primary objective was to demonstrate the tolerability of GVS protocol similar to tDCS treatment parameters (duration, intensity), in relation to the symptoms of motion sickness. Our results confirm that GVS induced motion sickness symptoms (Quinn et al., 2015). According to the neural mismatch model proposed by Reason and Brand (1975), sensory conflict induced by GVS leads to vegetative symptoms (Reason, 1978). GVS creates a sensory conflict between the information from an illusory lateral pulse of linear and angular accelerations and other sensory inputs from proprioception and vision (Severac, 1992). In the present study, vegetative disturbances rated by the participants appeared only for the double-duration session and provoked a low level of motion sickness symptoms (Paillard et al., 2014).

The secondary objective was to test the effect of GVS on anxiety. The present study demonstrates a significant decrease in anxiety level in a student population after a single-duration session of GVS. This result corroborates with similar findings that natural vestibular stimulation can induce a modification of mood state and anxiety (Winter et al., 2012, 2013; Kumar et al., 2016). In 2016, Kumar et al. (2016) demonstrated an improvement of anxiety state (STAI Y-A) and anxiety trait (STAI Y-B; Spielberger et al., 1970) in student population after a swinging protocol. Moreover, vestibular stimulation due to swinging can provoke immediate effects on mood state (Winter et al., 2012). Literature demonstrates that women can show cyclical changes in affect (Dennerstein and Burrows, 1979). Nevertheless, the present study demonstrated no effect of gender on anxiety measure. The positive effect of GVS on anxiety did not differ according to gender.

No significant result was obtained for the anxiety level during the double-duration session. This result indicates that the impact of GVS on anxiety is not duration dependent. In most studies, the improvement of emotional control occurs after 20 min of stimulation (Utz et al., 2010; Yokoi et al., 2017).

The modification of cortical excitability is a possible explanation to the positive effect of GVS on anxiety. In tDCS protocols, the application of direct current directly to the scalp induces regional changes in cortical excitability (Bennabi and Haffen, 2018). Beneficial effects of electric stimulation are due to modifications in synaptic connections. A possible hypothesis is the sedative effect of GVS on arousal state. Midbrain reticular formation is implicated in arousal and it’s electrical stimulation suppresses metabolic activity of vestibular nuclei in experimental animals (Gonzalez-Lima, 1987). Therefore, we might suppose that vestibular stimulation at low level is sedative, by working in antagonism to the ascending reticular activating system for arousal. Thereby, the reduced score in anxiety scale found might be explained by a decrease in arousal state. More researches about the impact of GVS are necessary to understand underlying neural mechanisms.

This research is a preliminary study of the possibility of using GVS in clinical applications. Some methodological limits should be discussed. Trait anxiety (STAI Y-B) was not assessed. This measure refers to stable interpersonal differences in anxiety propensity and will allow categorizing our population. Additionally, it seems interesting to complete the visual analog scale form measure with anxiety state test (STAI Y-A; Spielberger et al., 1970). Further research must include anxiety trait and state evaluations. In addition, forthcoming studies should increase the sample size in order to confirm these preliminary results.

After cortical stimulation, as with tDCS, plastic changes are detectable if the stimulated area is activated by a concurrent task execution (Trumbo et al., 2016; Pisoni et al., 2018). According to the literature and our present observations, it would be interesting to conduct another experimental protocol applying 20 min of stimulation in combination with a simultaneous affective task to promote cortical plasticity.

In conclusion, our study seems to show that 38 min of GVS decrease the anxiety level of young healthy adults. We also confirm the tolerability of a 38 min of GVS. It is well adapted for use and displays several technical advantages for potential future use as a treatment for anxiety disorders. Like tDCS, GVS represents a promising non-invasive tool to modulate neuronal excitability (Bennabi and Haffen, 2018). These results suggest that further research is necessary to confirm the role of GVS in the treatment of anxiety disorders and to understand the underlying mechanisms.

## Ethics Statement

This study was carried out in accordance with the recommendations of CERSTAPS 2019-18-09-37 with written informed consent from all subjects. All subjects gave written informed consent in accordance with the Declaration of Helsinki. The protocol was approved by the CERSTAPS.

## Author Contributions

FP, PD, AG, NB and GQ participated sufficiently in this submission to take public responsibility for its content. FP performed the experiment. FP and NB analyzed the data, and all authors participated in drafting the final article. Submission has been approved by all authors.

## Conflict of Interest Statement

The authors declare that the research was conducted in the absence of any commercial or financial relationships that could be construed as a potential conflict of interest.

## Supplementary Material

TABLE S1 Anxiety score and Graybiel score for time and session factors for all participants.

## Abbreviations

ANOVA: analysis of variance
GVS: galvanic vestibular stimulation
tDCS: transcranial direct current stimulation.
