# Corrigendum: Impact of Galvanic Vestibular Stimulation on Anxiety Level in Young Adults

**DOI:** 10.3389/fnsys.2019.00057

**Published:** 2019-10-22

**Authors:** Florane Pasquier, Pierre Denise, Antoine Gauthier, Nicolas Bessot, Gaëlle Quarck

**Affiliations:** Normandie Université, Unicaen, Inserm, Comete, GIP Cyceron, Caen, France

**Keywords:** anxiety, galvanic vestibular stimulation, motion sickness, vestibular system, direct current (DC)

In the original article, there was an error. The ethical committee provided in the original article is incorrect.

A correction has been made to the **Materials and Methods** section, paragraph one:

“Twenty-two students from the University of Caen, Normandy, participated in this study (age: 21.90 ± 1.37 years; 10 females and 12 males). This study was carried out in accordance with the recommendations of CERSTAPS 2019-18-09-37 with written informed consent from all subjects. All subjects gave written informed consent in accordance with the Declaration of Helsinki. The protocol was approved by the CERSTAPS. We excluded individuals with vestibular disorders and those with current or prior psychiatric disorders. One participant stopped the protocol after the first session. For women, experiments were scheduled outside of the menstrual period in order to avoid any bias due to hormonal fluctuations (Dennerstein and Burrows, 1979). The sessions were carried out in the late afternoon, on the same day of the week, and at the same hour to avoid any diurnal or weekly anxiety variations (Haffen and Sechter, 2006), for a total of three randomized sessions, for three consecutive weeks. Before and after each stimulation, participants completed a Graybiel Scale form for motion sickness evaluation (Graybiel et al., 1968), and a 100 mm visual analog scale form (relaxed—tense; Abend et al., 2014) to evaluate mood and psychophysiological alterations (Zealley and Aitken, 1969; Winter et al., 2012, 2013).”

Additionally, a correction has been made to the **Ethics Statement**:

“This study was carried out in accordance with the recommendations of CERSTAPS 2019-18-09-37 with written informed consent from all subjects. All subjects gave written informed consent in accordance with the Declaration of Helsinki. The protocol was approved by the CERSTAPS.”

The authors apologize for this error and state that this does not change the scientific conclusions of the article in any way. The original article has been updated.
